# Civic Participation as a Promoter of Well-Being: Comparative Analysis among European Countries

**DOI:** 10.1007/s11205-022-02947-0

**Published:** 2022-06-10

**Authors:** Andrea Vega-Tinoco, Ana Isabel Gil-Lacruz, Marta Gil-Lacruz

**Affiliations:** 1 Department of Business Direction and Organization, University of Zaragoza, Zaragoza, Spain; 2 Department of Psychology and Sociology, University of Zaragoza, Zaragoza, Spain

**Keywords:** Welfare system, Europe, Civic engagement, Older adults, Wellbeing

## Abstract

Previous research on the direction of the relationship between civic participation and well-being has evidenced that civic participation is a promoter of well-being among older adults in Europe. Accordingly, the objective of the present study is to identify and analyze the differences between European welfare systems regarding both civic participation and well-being. For this purpose, a logistic multilevel regression analysis was performed as an empirical strategy, using the cross-sectional data from the 9 waves of the European Social Survey (2002–2018). Significant differences in well-being were observed, with Anglo-Saxon elders being the healthiest, and the Nordic the happiest and most satisfied with their lives. In contrast, Eastern European seniors reported the lowest levels of well-being. Also, Nordic countries are the most civically engaged, followed by the Continental and Anglo-Saxon, while Mediterranean and Eastern countries engage the least. However, the impact of civic participation on well-being is strongest for the Mediterranean countries, while its impact on satisfaction and happiness is weakest for the Nordic countries. The 3 models of the multilevel analysis indicate that civic participation has a positive impact on health, happiness and life satisfaction, and that this effect is quite robust. Also, by adding country-level macro variables to the model, it is possible to reduce the random effects and hence to better explain these international differences. Concisely, the impact of civic participation on the well-being of the elderly differs across nations and should therefore be considered by policy makers.

## Introduction

The scientific literature on the benefits of senior citizen engagement is extensive. For example, engaging in volunteer activities improves the quality of life of older adults by providing them with greater life satisfaction and mental and physical health (Pettigrew et al., 2019; see also Dávila & Díaz-Morales 2005, 2009; Ferrada & Zavala, 2014; Haski-Leventhal, 2009). Similarly, other studies evidence that civic engagement contributes to the well-being of the elderly (Serrat et al., 2017; Wray-Lake et al., 2019) by generating, for example, community ties (Villar & Serrat, 2014) or improving mental health through social connectedness (Saeri et al., 2018). Therefore, it is important that governments and policy makers aim for strategies that promote these types of participation in Europe. However, not all European countries share the same participation rates, nor the same results on their well-being. Thus, it is of great importance to understand the main differences between countries (or welfare systems) to better target and adjust public and social policies that promote such engagement, in order to achieve the highest possible levels of well-being.

Previous research with the same database (Vega-Tinoco et al., 2021) has found empirical evidence on the bidirectional relationship between civic participation and the well-being of the elderly in Europe, but also that the effect of civic participation on well-being is greater than vice versa. Once the direction of the relationship between these variables has been confirmed (for further details please see Vega-Tinoco et al., 2021), the main objective of the current work is to study the international differences between European welfare systems. For this purpose, we have employed multilevel regression analysis using data from the European Social Survey (ESS 2002–2018). Understanding these differences is relevant in order to identify whether civic participation affects the well-being of the elderly equally across the continent, or not, so that the public policies of each system can be more specifically targeted to encourage the participation of their population.

Thus, this study provides some contributions to the state of the art. The first is the determination of the differences regarding well-being and civic participation among welfare systems, from 2002 to 2018. The second is the analysis of well-being through three different indicators, which provide a broader perspective when identifying the potential determinants of well-being (Burns, 2019; VanderWeele et al., 2020). The third contribution refers to studying international differences at various levels, so that the weight of civic participation on well-being is determined separately, then with individual-level factors and, finally, by adding macro variables at the country level in order to better explain the well-being differences between welfare systems.

## Literature Review

Population aging in Europe shows a constant increasing trend, with 21% of the population being over 65 years old in 2020 and with an increment of 3 percentage points compared to the previous decade (Eurostat, 2021). For this reason, the governments of European countries and international organizations propose a series of actions and policies to mitigate the possible adverse effects of population aging, while encouraging those strategies that promote the active and healthy aging of their citizens. Some examples are the White Paper on Active Aging that reflects the initiative of the Spanish government to guide national policies aimed at improving the quality of life of its elderly (Instituto de Mayores y Servicios Sociales [IMSERSO], 2018); the United Nations Decade of Healthy Ageing (2021–2030) that calls for coordinated and collaborative actions in order to improve the lives of the older adults, their families and their communities; or the Strategic Framework for Mainstreaming Ageing suggested by the UN Economic Commission for Europe (UNECE) with the aim of systematically considering and integrating aging within public policies (United Nations [UN], 2021). In addition, the COVID-19 pandemic has demonstrated the need to consider aging transversally due to the (serious) repercussions that gaps within existing systems, policies and services may have on the population (UN, 2020).

Active aging is often contextualized from a broad perspective of biological, psychological and social well-being. The *Active ageing policy framework* published by the World Health Organization (WHO, 2002) identifies *active aging* as a process by which people´s opportunities of health, participation and security are optimized with the intention of improving their quality of life as they grow older. This model includes autonomy, independence and quality of life as key aspects.

The active aging notion was first attributed to Havighurst (1961) and later complemented with the concept of successful aging, which he described in terms of happiness and satisfaction with life. The author pointed out that active or successful aging is a process that should be carried out throughout the entire life cycle in order to reach a happy old age, through considering those conditions that lead a person to their highest possible level of satisfaction. Rowe & Kahn (1997) also proposed a model of successful aging that goes beyond the absence of disease and the maintenance of functional capacity, transcending towards their combination with an active participation throughout life. The same authors highlight that active aging identifies as important those activities that are of special relevance to the person, that create social value and that generate interpersonal relationships (see also Petretto et al., 2016; Urrutia, 2018). Social participation greatly influences quality aging (Morawski et al., 2020).

Civic engagement, understood as those actions performed by ordinary citizens that seek to influence the processes that affect them and their environment (Thomassen, 2003; Grasso et al., 2019), is a democratic symbol of the voice and awareness of citizens and the space of freedom in which to exercise their rights (McBride et al., 2006). Within this type of participation, and in accordance with the ESS (Thomassen, 2003; ESS, 2018), the following are considered civic activities: voluntary involvement in political and non-political organizations, signing petitions, wearing campaign badges, contacting politicians, boycotting products and demonstrating publicly.

Civic participation has a positive impact on the well-being of the elder European population. By means of a pseudo-panel, previous research (Vega-Tinoco et al., 2021) has evidenced that, although there is a bidirectional relationship between civic participation and well-being, the impact that past participation has on present health and happiness is stronger than vice-versa. Accordingly, among the well-being indicators, the following 3 stand out: health, happiness and life satisfaction, which have been considered in the present study as dependent variables (Helliwell & Putnam, 2004; WHO, 2002; Petretto et al., 2016; VanderWeele et al., 2020). This subjective approach to well-being represents a good measure of people’s actual quality of life (Huppert et al., 2005; VanderWeele et al., 2020). Furthermore, using several indicators allows a broader perspective on the factors that influence the well-being of senior Europeans (Burns, 2019; VanderWeele et al., 2020).

However, European citizenry is not homogeneous, nor are its rates of civic participation and well-being. Gil-Lacruz & Marcuello (2012) highlight that volunteering rates vary between welfare systems, being GDP per capita and government expenditures on social issues factors that reinforce them. The authors also highlight that “national contextual data reduces the unobserved variability among countries, especially among countries with different welfare systems; …” (Gil-Lacruz & Marcuello, 2012, p. 380). Similar to volunteering, it seems feasible that high variability in civic participation rates may be found across countries and that these rates may be higher as the economic development of the country increases (Morawski et al., 2020). Thus, it would be expected that citizens in wealthier countries would also be the most engaged in this type of participation. According to the European Parliament Civic Engagement Report (2020) civic engagement rates (measured by being involved with civil society organisations) vary among European countries from 23% in Hungary and 30% in Bulgaria to 66% in the Netherlands, 68% in Sweden and 70% in Denmark.

Likewise, given that these types of activities are positively correlated with quality of life (Haski-Leventhal, 2009; Morawski et al., 2020; Vega-Tinoco et al., 2021) it could also be expected that the impact of volunteering would be higher in those countries with higher rates of participation. However, in the study by Morawski et al., (2020) the relationship between volunteering and the economic development of a country has an inverted U-shape, so that the correlation between volunteering and quality of life is weak in those countries with the highest (Denmark, Switzerland, and Belgium) and lowest (Poland, Greece, the Czech Republic, and Spain) rates of volunteering, while the correlation is strong in countries with medium levels of volunteering (Austria, Italy, and Israel). Again, the relationship between civic participation and the well-being of older adults could be expected to be similar.

In terms of well-being, variability is also found in the levels of health, satisfaction and happiness across European countries. According to data from the World Database of Happiness (Veenhoven, 2020) countries with Nordic welfare systems are the most satisfied with life, followed by the Continental and Anglo-Saxon, while Eastern and Mediterranean countries report the lowest values (OECD, 2021). Additionally, Anglo-Saxon countries present the best health perceptions, followed by Nordic, Continental, Mediterranean and Eastern countries (OECD, 2021). On the other hand, according to Akaeda (2021), several studies provide evidence that civic participation reduces the risk of mortality and poor self-reated health in Southern and Western European countries (such as the United Kingdom and Germany), while others suggest no association between civic participation and health in Canada and Japan.

These differences may be due to a series of sociodemographic factors at the individual level, as well as contextual factors at the country level. From the point of view of the sociology of aging, it has been argued that organizations and policy makers have placed the responsibility for active and healthy aging on the individual. However, the active aging of a person does not depend solely on his or her responsibility for his/her own lifestyle, but also on the social context in which he or she develops, being of particular importance the inequality in the distribution of wealth and resources (Hayes, 2021), factors directly related to the individual’s country of residence. For example, several studies have confirmed the positive impact of social capital on health at both the individual and ecological levels (Ehsan et al., 2019), but this impact may also be modified by the actions of public social institutions within each welfare system and by the level of equity in the distribution of income and wealth within countries (Islam et al., 2006). Thus, the positive relationship between social capital and health varies from country to country and appears to be stronger in those with greater economic inequality; that is, in less egalitarian countries, the level of social capital contributes to explaining differences in health between regions, while in more egalitarian countries, social capital does not play a significant role when it comes to clarifying such health differences (Islam et al., 2006; Ehsan et al., 2019).

Furthermore, public social expenditures may also be a moderator of such a relationship given that “some studies suggest that welfare provisions and policies may influence the health impact of social capital” (Akaeda, 2021, p.5). Moreover, the results of the study conducted by Akaeda (2021) indicate that this effect may be enhanced by welfare provisions, so it would be of great interest to study whether this is also the case for other well-being indicators. Thus, just as country characteristics modify the relationship between health and social capital — to which civic participation belongs in its structural dimension (Islam et al., 2006; Akaeda, 2021) — macro level variables may also modify the relationship between civic participation and health, happiness or life satisfaction and contribute to explaining international differences.

Therefore, in this paper we consider some macro variables. A commonly used metric is the level of national income determined by the gross domestic product (GDP) per capita. However, it is also necessary to take into account the distribution of such wealth. For this purpose, we have included two variables: the Gini coefficient of equivalised income and the public social expenditure rate. The Gini index is comprised between 0 and 1, where 1 is perfect inequality and 0 is perfect equality, and it is determined by the cumulative proportions of the population compared to the cumulative proportions of income they receive. Public social expenditure refers to relevant financial flows controlled by the government (such as cash benefits, direct provision of goods and services, and tax breaks with a social purpose), as a percentage of the GDP (OECD, 2016).

## Hypothesis

Based on the scientific literature, the hypotheses of the present research are as follows:

**H1**: Rates of civic participation and well-being vary according to European welfare systems: Nordic countries are the most participatory and have the highest well-being levels, while Eastern Europeans are the least participatory and have the lowest well-being.

### H2

The effect of civic participation on well-being is stronger in countries with medium participation rates, and weaker in countries with high and low participation rates.

## Database

The data used in this study have been extracted from the 9 available waves of the European Social Survey (ESS) from 2002 to 2018 (ESS 2002; 2004; 2006; 2008; 2010; 2012; 2014; 2016; 2018). For each wave we have created dummy variables to control for time effects. Additionally, we have chosen the 14 European countries with sufficient information for all waves and assigned them to one of the following 5 welfare systems to control for geographical effects at country level and welfare system level: Belgium, France, Germany, Netherlands and Switzerland (Continental welfare system); Finland, Norway and Sweden (Nordic welfare system); Hungary and Poland (Eastern welfare system); Ireland and the United Kingdom (Anglo-Saxon welfare system); Portugal and Spain (Mediterranean welfare system). Also, since the study is oriented towards the elderly, we have considered people aged 50 years and older who reside in the aforementioned countries. The final sample consists of 114,331 individuals.

From the ESS we have also obtained the sociodemographic variables (at the individual level) related to: age, gender, legal marital status, highest level of education, labor situation (main activity in the last 7 days), income level (the feeling about one’s household income nowadays) and size of residential area (living in a city, town or countryside). Regarding civic participation, respondents were asked whether they had carried out any of the following activities in the last 12 months in order to improve things in their country: contacted a politician, were involved in a political party or another organization or association, worn a campaign badge, signed a petition, publicly demonstrated, or boycotted any products. The variable *CivicParticipation* was coded 1 if the person had performed any of the aforementioned activities, and 0 if otherwise.

Concerning well-being, variables correspond to the following ESS questions. *Health* is measured by “How is your health in general?” with a scale between 1 and 5, where 1 means that the person considers their health to be “Very good” and 5 as “Very bad”. This scale has been recoded (1 = “Very bad” to 5 = “Very good”) to facilitate the interpretation of results. Regarding *Happiness* and *LifeSatisfaction*, the questions state: “Taking all things together, how happy would you say you are?“ and “All things considered, how satisfied are you with your life as a whole nowadays?“. Interviewees responded using an 11-point scale between “Extremely unhappy/unsatisfied” and “Extremely happy/satisfied”.

Furthermore, national information on the macro variables GDP per capita and social expenditure was extracted from the OECD Health Statistics 2020, while the Gini index was drawn from the Eurostat Database. For additional information on these variables, please refer to Table 1.

**Table 1:** Description of variables

Variable	Description	Welfare system	Mean^d^	Std. Dev.	Variation 2002–2018
Gross Domestic Product (GDP)^a^	US Dollars ($) per capita, constant prices, constant purchasing power parity, base year 2015, OECD	Anglo-Saxon	49538.33	11008.61	41.36%
		Continental	48553.98	8331.59	17.58%
		Eastern	23884.00	3716.34	63.84%
		Mediterranean	32654.44	2875.92	10.78%
		Nordic	49580.00	7467.23	16.19%
Gini^ac^	Coefficient of equivalised disposable income, EU-SILC survey Eurostat	Anglo-Saxon	0.32	0.02	-5.60%
		Continental	0.28	0.02	5.33%
		Eastern	0.30	0.03	-4.72%
		Mediterranean	0.34	0.02	-5.22%
		Nordic	0.25	0.01	4.72%
Social expenditure^a^	From public source, in percentage of Gross Domestic Product, OECD	Anglo-Saxon	0.19	0.03	4.72%
		Continental	0.23	0.06	2.90%
		Eastern	0.21	0.01	-7.60%
		Mediterranean	0.23	0.02	17.78%
		Nordic	0.25	0.03	8.60%
Civic participation^b^	Percentage of people who have answered “yes” to involving in any civic activity in order “improve things in [their country] or help prevent things from going wrong” in the past 12 months	Anglo-Saxon	0.47	0.50	-3.71%
		Continental	0.53	0.50	16.45%
		Eastern	0.17	0.37	0.55%
		Mediterranean	0.27	0.44	100.93%
		Nordic	0.67	0.47	19.74%
Health^b^	Percentage of people who have reported being in good health or very good health when asked: “How is your health in general?“	Anglo-Saxon	0.66	0.47	1.25%
		Continental	0.58	0.49	7.75%
		Eastern	0.31	0.46	66.27%
		Mediterranean	0.36	0.48	24.55%
		Nordic	0.60	0.49	15.36%
Happiness^b^	Percentage of people who have reported being happy or extremely happy when asked: “Taking all things together, how happy would you say you are?“	Anglo-Saxon	0.34	0.47	-17.09%
		Continental	0.27	0.44	17.20%
		Eastern	0.17	0.37	28.14%
		Mediterranean	0.18	0.39	72.52%
		Nordic	0.37	0.48	12.94%
Life satisfaction^b^	Percentage of people who have reported being satisfied or extremely satisfied with their lives when asked: “All things considered, how satisfied are you with your life as a whole nowadays?“	Anglo-Saxon	0.29	0.45	-5.87%
		Continental	0.27	0.44	13.91%
		Eastern	0.14	0.35	22.60%
		Mediterranean	0.14	0.35	112.84%
		Nordic	0.40	0.49	13.74%

a. Units of analysis are countries

b. Units of analysis are individuals

c. Missing data in the database have been filled by calculating the values based on the increase/decrease of previous years

d. Differences between groups are significant (Kruskal-Wallis test)

## Empirical Framework

In a preliminary analysis of the database it was observed that civic participation is positively correlated with health, happiness and life satisfaction. Therefore, in order to facilitate the interpretation of the results, we decided to keep only the high levels of well-being (perceiving one’s health to be “Good or Very good”, being “Happy or Extremely happy” and being “Satisfied or Extremely satisfied with life”). Thus, it resulted in our dependent variables *Health*, *Happiness* and *LifeSatisfaction* to be binary (1 = high well-being; 0 = otherwise).

Seeking to understand the differences in well-being among countries while considering civic participation as the explanatory variable of interest (which is the main objective of this work), multilevel mixed-effects logistic regression models have been used as an empirical strategy. To this end, we used the “STATA 14” software (command: xtmelogit). Given that multilevel models simultaneously examine the effects of both group and individual level variables on individual level outcomes, they are appropriate when the data comprise nested sources of variability, that is, lower level units (*i*) nested within higher group levels (*j*) (Diez-Roux, 2002; Iglesias-Pascual et al., 2021). In our case, at the individual level we find the persons interviewed in the ESS (*i* = 114,331) who at the same time belong to *j* = 14 countries. Thus, for each of the well-being indicators (dependent variables ${Health}_{ij}$, ${Happiness}_{ij}$ and ${LifeSatisfaction}_{ij}$) the equation is represented as:

1 $${WellbeingIndicator}_{ij}= \stackrel{-}{\beta }+ {u}_{j}+{e}_{ij}$$

where the variance components are distinguished: $\stackrel{-}{\beta }$ represents the fixed effects (i.e. factors that are common across countries) and ${u}_{j}$ represents the random effects (i.e. factors that may vary randomly across countries). ${e}_{ij}$ is the error component. This model assumes that both the random effects ${u}_{j}$ and the error term ${e}_{ij}$ are independent, are distributed normally with mean 0 and variance ${\sigma }^{2}$, and also that all the ${e}_{ij}$ are independent from one another.

Additionally, a set of individual factors ($K-1$ variables and a constant) corresponding to the fixed effects have been included within a ${X}_{ij}^{{\prime }}$ vector. Then, Eq. (2) becomes:

2 $${WellbeingIndicator}_{ij}= {X}_{ij}^{{\prime }}{\beta }_{j}+ {u}_{j}+{e}_{ij}$$

Where X comprises K regressors and ${\beta }_{j}$ is the country specific effect of the individual level factors.

Lastly, the ${\beta }_{j}$ coefficients are parametrized by adding L national explanatory variables, thus the fixed effects also depend on these macro variables, in addition to the individual level variables and the constant. As a result, Eq. (3) is expressed as:

3 $${WellbeingIndicator}_{ij}= {X}_{ij}^{{\prime }}{\stackrel{-}{\beta }}_{j}+ {u}_{j}+{e}_{ij}$$

Based on these general models, this work comprehends 3 models for each wellbeing indicator, including different factors in order to reduce the influence of random effects. Model 1 includes *civic participation* as the independent variable of interest, along with time dummy variables. Subsequently, in Model 2, sociodemographic explanatory factors at the individual level, such as age, gender, marital status, education level, working status, income level and area of residence, are incorporated so as to reduce the unobserved term. Finally, Model 3 introduces macro variables at the country level, such as GDP per capita, Gini index and social expenditure rate. Variables representing welfare systems were also included in Model 3, as contextual data, in order to determine whether the international differences in senior well-being were strongly influenced by the welfare system they live in.^1^ Models 2 and 3 provide confirmation of whether the impact of civic participation on well-being is mitigated when controlling for individual- and country-level factors, and whether differences in well-being among countries are given by both individual-level and country-level factors. Additionally, we have performed an analysis of variance to better examine these differences. Given that the ANOVA method could not be employed since not all welfare groups were normally distributed, we opted for its nonparametric equivalent, the Kruskal-Wallis test.

## Results

### Descriptive Statistics

In addition to presenting the description of the most relevant variables for this work, Table 1 exhibits the descriptive results for each welfare system, including the mean and standard deviation of each variable, as well as its percentage change between the years 2002 and 2018. These descriptive results are presented below, first for the macro variables at the country level (GDP, Gini, social expenditure), and then for the variables at the individual level (civic participation and well-being indicators).

The table shows that, on average, the countries belonging to the Nordic, Anglo-Saxon and Continental systems have the highest GDP per capita, respectively, and also hold quite similar GDP amounts. With a marked difference, they are followed by the Mediterranean countries and finally the Eastern countries, although it is worth noting that the latter are the ones that have achieved the highest percentage variation, with an increase in their GDP per capita of approximately 64%. The Anglo-Saxon system has also obtained a significant variation (+ 41%), whereas the increases of the other systems have not been quite as high: Continental (+ 18%), Nordic (+ 16%) and Mediterranean (+ 11%).

Regarding the Gini index of income inequality, there is evidence that the Nordic (0.25) and Continental (0.28) countries are the most equitable, followed by the Eastern (0.30), Anglo-Saxon (0.32) and Mediterranean (0.34) countries, respectively. However, this gap tends to become smoother over time, as the most unequal countries improve their Gini index between 2002 and 2018, and the most equitable countries worsen this ratio. Additionally, the countries with the highest percentage of social expenditure from public sources are the Nordic, amounting to approximately 25% of their GDP, followed by the Continental (23%), Mediterranean (23%), Eastern (21%) and Anglo-Saxon (19%), respectively. All welfare systems have increased their social expenditure between 2002 and 2018, being the Mediterranean those to increase it the most (+ 18%) and the Continental the least (+ 3%), except for the Eastern nations, whose social expenditure has reduced by 7.6%.

As for the individual-level variables, 67% of Nordic residents have engaged in some civic participation activity in the past year, as have 53% of residents in Continental countries and 47% in Anglo-Saxon countries. In contrast, only 27% and 17% of senior citizens have participated civically in Mediterranean and Eastern countries, respectively. However, almost all nations have increased their civic participation between 2002 and 2018, the most remarkable case being that of the Mediterranean with a positive variation of almost 101%, and contrasting with the Anglo-Saxon whose participation has decreased by 4%.

Regarding well-being indicators, older adults in the Nordic countries express the highest happiness and life satisfaction (with a significant difference in satisfaction compared to the other welfare systems), followed by the Anglo-Saxon and then the Continental. However, it is the Anglo-Saxon who express the best health, followed by the Nordic and the Continental. In contrast, the Mediterranean and Eastern countries have the lowest well-being scores, with the Eastern scoring the lowest, although closely followed by the Mediterranean. However, it is also these countries that show the largest increases in well-being between 2002 and 2018, most notably for the increase in life satisfaction of Mediterranean residents (+ 113%), as well as in their happiness (+ 73%). It is also worth noting that all welfare systems have presented an increase in their well-being levels, with the exception of the Anglo-Saxon, whose happiness and life satisfaction have decreased by 17% and 6%, respectively.

Table 2 shows the differences among European welfare systems regarding the correlation between civic participation and well-being indicators. This correlation is stronger in those welfare systems with high and low participation rates, while the relation is weaker in countries with medium levels of participation. It is noteworthy that the correlation between participation and well-being is stronger for Mediterranean countries than for all other groups, especially when it comes to the relationship between participation and health and, furthermore, given that their self-reported health levels are among the lowest. On the other hand, the weakest correlation scores correspond to the Anglo-Saxon countries, though their health average is the highest. Similarly, even though the Nordic report the highest averages of happiness and life satisfaction, the correlation between participation and these indicators is quite low. Additionally, in all welfare systems civic participation is more strongly related to health than to the other indicators of well-being.

**Table 2:** Correlation between Civic Participation and Well-being indicators. Differences among European welfare systems

	Anglo-Saxon	Continental	Eastern	Mediterranean	Nordic
Civic Participation - Health	0.05	0.08	0.08	0.14	0.09
Civic Participation - Happiness	0.03	0.04	0.03	0.08	0.03
Civic Participation - Life Satisfaction	0.03	0.05	0.04	0.09	0.02

All values correspond to Spearman pairwise correlations. All values are significant at p < 0.01

Units of analysis are individuals

### Multilevel Logistic Regression Analysis

As mentioned before, 3 models have been carried out for every well-being indicator, each model including different factors in order to explain the variability of the random effects. Like this, the unobserved term is considerably reduced from Model 1 to Model 2 once the sociodemographic explanatory factors at the individual level are incorporated. Likewise, a significant reduction in the σ^2^_u_ term is observed in Model 3 for all dependent variables. Additionally, all models present an adjustment of p < 0.05, and the Kruskal-Wallis test suggests that the differences between groups are significant. The main results of the 3 models for each well-being indicator are presented below.

*Health*.

In Model 1, the impact of civic participation on health is substantial. A positive association between health and the most recent years is also observed. In Model 2, the impact of civic participation is significantly reduced, although it is still positively correlated with health. The relationship between health and the most current years remains positive, while it is negatively related to age. Additionally, being male, being married, living in a city and working increase the probability of having good health. Also, the level of education and the level of income are positively related to health.

The results of Model 2 for both civic participation and the socio-demographic fixed effects are maintained in Model 3, which provides evidence of the robustness of the model, with the added bonus that the inclusion of the country level factors helps to better explain the health differences between countries. It is also observed that a country’s GDP has a practically null effect on health and, furthermore, that as GDP increases, its impact on health becomes even smaller. In addition, an increase in a country’s level of economic inequality increases the probability that its inhabitants perceive their health to be good; although, as inequality increases, this effect becomes linear given that the quadratic effect of the Gini index is nearly zero (i.e., the growth rate remains slightly declining). In contrast, the national social expenditure made by the public sector is negatively correlated with health whereas, similar to the Gini index, its exponential effect is linear. Finally, residing in an Anglo-Saxon country considerably increases the probability of being healthier, compared to living in a Mediterranean country.

(See Table 3)

**Table 3:** Logistic multilevel regression results for independent variable Health (xtmelogit)

Health	Model 1		Model 2		Model 3	
	Coef.	p	Coef.	p	Coef.	p
*Fixed effects*						
Intercept	-0.03	0.86	1.39	0.00	-1.76	0.04
Civic participation	0.37	0.00	0.09	0.00	0.09	0.00
Year 2002	-0.12	0.00	-0.11	0.00	-0.04	0.34
Year 2004	-0.08	0.00	-0.06	0.04	-0.01	0.77
Year 2006	-0.06	0.02	-0.05	0.10	-0.03	0.39
Year 2008^a^	--	--	--	--	--	--
Year 2010	0.00	0.91	0.01	0.85	-0.01	0.80
Year 2012	0.06	0.02	0.07	0.01	0.06	0.06
Year 2014	0.06	0.02	0.03	0.21	0.00	0.99
Year 2016	0.07	0.00	0.00	0.99	-0.06	0.10
Year 2018	0.16	0.00	0.08	0.01	0.02	0.61
Age 50–64	--	--	0.18	0.00	0.19	0.00
Age 65-79^a^	--	--	--	--	--	--
Age 80+	--	--	-0.41	0.00	-0.41	0.00
Male^a^	--	--	--	--	--	--
Female	--	--	-0.07	0.00	-0.07	0.00
Married^a^	--	--	--	--	--	--
Single	--	--	-0.12	0.00	-0.12	0.00
Divorced	--	--	-0.09	0.00	-0.09	0.00
Widow	--	--	-0.15	0.00	-0.15	0.00
Primary or less	--	--	-0.59	0.00	-0.59	0.00
Secondary	--	--	-0.25	0.00	-0.25	0.00
Tertiary^a^	--	--	--	--	--	--
Worker^a^	--	--	--	--	--	--
Unemployed	--	--	-0.37	0.00	-0.37	0.00
Retired	--	--	-0.67	0.00	-0.67	0.00
Housework	--	--	-0.52	0.00	-0.52	0.00
Disabled	--	--	-2.62	0.00	-2.62	0.00
Other	--	--	-0.38	0.00	-0.38	0.00
Low income	--	--	-1.08	0.00	-1.08	0.00
Middle income	--	--	-0.46	0.00	-0.46	0.00
High income^a^	--	--	--	--	--	--
City^a^	--	--	--	--	--	--
Town	--	--	-0.05	0.00	-0.05	0.00
Countryside	--	--	-0.03	0.06	-0.03	0.05
GDP	--	--	--	--	0.00	0.00
GDP^2^	--	--	--	--	-0.00	0.00
Gini	--	--	--	--	0.16	0.00
Gini^2^	--	--	--	--	-0.00	0.00
Social expenditure	--	--	--	--	-0.07	0.01
Social expenditure^2^	--	--	--	--	0.00	0.00
Mediterranean^a^	--	--	--	--	--	--
Anglo-Saxon	--	--	--	--	0.78	0.02
Continental	--	--	--	--	0.23	0.41
Eastern	--	--	--	--	-0.17	0.60
Nordic	--	--	--	--	0.20	0.51
*Random effect*						
σ^2^_u_ Standard Deviation	0.59 (0.11)		0.53 (0.10)		0.32 (0.06)	
Kruskal-Wallis test	0.00		0.00		0.00	
Model adjustment	0.00		0.00		0.00	

^a^ Reference variable

*Happiness*.

The impact of civic participation on happiness remains fairly stable throughout models, which gives us the idea that it is quite robust. In all 3 models, we observe that the most current years are more strongly correlated with the happiness of older people (compared to 2008, as the reference year), with lower probability of being happy in the years around 2008. Similar to the health results, the coefficients of the socio-demographic variables in Model 2 are maintained in Model 3. Happiness is positively correlated with age and with the income level, while it is negatively correlated with the education level. Also, being a woman, living in the countryside and being married increase the probability of being happy, as opposed to being a man, living in the city and having another marital status. On one hand, being retired and doing housework increase the probability of happiness compared to being a worker, while on the other, being unemployed or having a disability reduces it. As with health, national social expenditure is negatively correlated with the happiness of the elderly, while its quadratic effect becomes linear as social expenditure increases. In addition, the probability of happiness increases when residing in a Continental, Anglo-Saxon or Nordic country, respectively, compared to Mediterranean countries.

(See Table 4)

**Table 4:** Logistic multilevel regression results for independent variable Happiness (xtmelogit)

Happiness	Model 1		Model 2		Model 3	
	Coef.	p	Coef.	p	Coef.	p
*Fixed effects*						
Intercept	-1.21	0.00	-0.74	0.00	0.46	0.60
Civic participation	0.14	0.00	0.12	0.00	0.11	0.00
Year 2002	0.12	0.00	0.11	0.00	-0.01	0.89
Year 2004	0.09	0.00	0.08	0.01	0.01	0.72
Year 2006	0.01	0.66	0.01	0.82	-0.03	0.32
Year 2008^a^	--	--	--	--	--	--
Year 2010	-0.01	0.73	0.02	0.49	0.06	0.09
Year 2012	0.16	0.00	0.19	0.00	0.22	0.00
Year 2014	0.13	0.00	0.14	0.00	0.18	0.00
Year 2016	0.28	0.00	0.26	0.00	0.34	0.00
Year 2018	0.31	0.00	0.30	0.00	0.42	0.00
Age 50–64	--	--	-0.13	0.00	-0.13	0.00
Age 65-79^a^	--	--	--	--	--	--
Age 80+	--	--	0.11	0.00	0.11	0.00
Male^a^	--	--	--	--	--	--
Female	--	--	0.21	0.00	0.21	0.00
Married^a^	--	--	--	--	--	--
Single	--	--	-0.62	0.00	-0.62	0.00
Divorced	--	--	-0.49	0.00	-0.50	0.00
Widow	--	--	-0.65	0.00	-0.65	0.00
Primary or less	--	--	0.23	0.00	0.23	0.00
Secondary	--	--	0.05	0.01	0.05	0.01
Tertiary^a^	--	--	--	--	--	--
Worker^a^	--	--	--	--	--	--
Unemployed	--	--	-0.21	0.00	-0.21	0.00
Retired	--	--	0.07	0.00	0.07	0.00
Housework	--	--	0.08	0.01	0.08	0.01
Disabled	--	--	-0.19	0.00	-0.19	0.00
Other	--	--	0.28	0.00	0.28	0.00
Low income	--	--	-1.10	0.00	-1.09	0.00
Middle income	--	--	-0.58	0.00	-0.58	0.00
High income^a^	--	--	--	--	--	--
City^a^	--	--	--	--	--	--
Town	--	--	-0.00	0.85	-0.00	0.88
Countryside	--	--	0.10	0.00	0.10	0.00
GDP	--	--	--	--	-0.00	0.12
GDP^2^	--	--	--	--	-0.00	0.69
Gini	--	--	--	--	0.08	0.13
Gini^2^	--	--	--	--	-0.00	0.12
Social expenditure	--	--	--	--	-0.15	0.00
Social expenditure^2^	--	--	--	--	0.00	0.00
Mediterranean^a^	--	--	--	--	--	--
Anglo-Saxon	--	--	--	--	0.90	0.01
Continental	--	--	--	--	0.61	0.03
Eastern	--	--	--	--	-0.13	0.70
Nordic	--	--	--	--	1.18	0.00
*Random effect*						
σ^2^_u_ Standard Deviation	0.47 (0.09)		0.39 (0.07)		0.32 (0.06)	
Kruskal-Wallis test	0.00		0.00		0.00	
Model adjustment	0.00		0.00		0.00	

^a^ Reference variable

*Satisfaction with life*.

In Model 1, civic participation has a positive impact on life satisfaction and, from 2012 onwards, the probability of satisfaction is higher with respect to 2008. The impact of civic participation is slightly reduced in Model 2 and is maintained in Model 3, therefore considered as quite robust. Also, the coefficients corresponding to the years 2012–2018 increase, yet maintaining the same trend. In addition, the effect of the sociodemographic factors is alike in Models 2 and 3. As with happiness, life satisfaction is positively correlated with age and income level, and negatively correlated with education level. Also, the likelihood of being satisfied with life increases if one is female, married, or lives in a town or in the countryside, as opposed to being male, unmarried, and living in the city. In addition, being retired or doing housework also increases the possibility of satisfaction compared to being a worker, while being unemployed or having a disability reduces it.

In reference to the macro variables, the impact of GDP on life satisfaction is practically null. On the other hand, life satisfaction is positively correlated with the level of inequality and negatively correlated with the level of national social expenditure. In all cases, the exponential effect of the macro variables becomes linear as they increase. In addition, living in a Continental, Anglo-Saxon or Nordic country considerably increases the probability of being satisfied with life, respectively, compared to living in a Mediterranean country.

(See Table 5)

**Table 5:** Logistic multilevel regression results for independent variable Life Satisfaction (xtmelogit)

Life satisfaction	Model 1		Model 2		Model 3	
	Coef.	p	Coef.	p	Coef.	p
*Fixed effects*						
Intercept	-1.30	0.00	-0.80	0.00	-0.21	0.82
Civic participation	0.16	0.00	0.13	0.00	0.13	0.00
Year 2002	0.02	0.43	0.02	0.45	-0.09	0.03
Year 2004	0.05	0.10	0.05	0.10	-0.01	0.85
Year 2006	0.01	0.73	0.01	0.71	-0.03	0.36
Year 2008^a^	--	--	--	--	--	--
Year 2010	0.03	0.26	0.07	0.03	0.14	0.00
Year 2012	0.24	0.00	0.28	0.00	0.35	0.00
Year 2014	0.10	0.00	0.10	0.00	0.20	0.00
Year 2016	0.22	0.00	0.18	0.00	0.32	0.00
Year 2018	0.25	0.00	0.23	0.00	0.39	0.00
Age 50–64	--	--	-0.17	0.00	-0.17	0.00
Age 65-79^a^	--	--	--	--	--	--
Age 80+	--	--	0.08	0.00	0.08	0.00
Male^a^	--	--	--	--	--	--
Female	--	--	0.21	0.00	0.21	0.00
Married^a^	--	--	--	--	--	--
Single	--	--	-0.36	0.00	-0.35	0.00
Divorced	--	--	-0.29	0.00	-0.29	0.00
Widow	--	--	-0.37	0.00	-0.37	0.00
Primary or less	--	--	0.15	0.00	0.14	0.00
Secondary	--	--	0.03	0.14	0.03	0.14
Tertiary^a^	--	--	--	--	--	--
Worker^a^	--	--	--	--	--	--
Unemployed	--	--	-0.38	0.00	-0.38	0.00
Retired	--	--	0.13	0.00	0.13	0.00
Housework	--	--	0.14	0.00	0.14	0.00
Disabled	--	--	-0.37	0.00	-0.36	0.00
Other	--	--	0.27	0.00	0.28	0.00
Low income	--	--	-1.42	0.00	-1.42	0.00
Middle income	--	--	-0.75	0.00	-0.75	0.00
High income^a^	--	--	--	--	--	--
City^a^	--	--	--	--	--	--
Town	--	--	0.03	0.12	0.03	0.12
Countryside	--	--	0.16	0.00	0.16	0.00
GDP	--	--	--	--	-0.00	0.06
GDP^2^	--	--	--	--	-0.00	0.99
Gini	--	--	--	--	0.10	0.10
Gini^2^	--	--	--	--	-0.00	0.07
Social expenditure	--	--	--	--	-0.10	0.00
Social expenditure^2^	--	--	--	--	0.00	0.05
Mediterranean^a^	--	--	--	--	--	--
Anglo-Saxon	--	--	--	--	0.98	0.02
Continental	--	--	--	--	0.95	0.01
Eastern	--	--	--	--	-0.07	0.88
Nordic	--	--	--	--	1.65	0.00
*Random effect*						
σ^2^_u_ Standard Deviation	0.66 (0.13)		0.55 (0.10)		0.41 (0.08)	
Kruskal-Wallis test	0.00		0.00		0.00	
Model adjustment	0.00		0.00		0.00	

^a^ Reference variable

## Discussion

Once the direction of the relationship between civic participation and well-being has been studied and having evidenced that civic participation, and its different activities, are promoters of well-being among older adults in Europe (for more details see Vega-Tinoco et al., 2021), this paper studies the influence of civic participation on well-being by controlling for fixed and random effects through a hierarchical database at three levels (individual, nation and welfare system). Among the main results, we find that civic participation has a positive impact on health, happiness and life satisfaction, and the fact that the coefficients remain fairly stable throughout the 3 models of the multilevel analysis indicates that the effect is quite robust. Also, by adding country level factors to the model it is possible to reduce the random effects and hence to better explain the international differences.

Regarding well-being, Nordic countries report the highest levels of happiness and life satisfaction, followed by the Anglo-Saxon and then the Continental. On the other hand, it is the Anglo-Saxons who express the highest health scores, followed by the Nordic and Continental countries. In contrast, the Eastern countries obtained the lowest scores, although closely followed by the Mediterranean; yet it is also these two groups of countries that have obtained the largest increases in well-being between 2002 and 2018. These results are partly similar to those found by Requena (2010), whose study indicates that countries in the Eastern and Mediterranean systems report the lowest subjective well-being, while the Anglo-Saxons show the highest levels. However, the differences between studies may lie in the fact that Requena includes non-European countries in the Anglo-Saxon system such as the United States, Canada and Australia, while in the Continental system, only Germany is included. Requena (2010) also suggests that this subjective well-being “correlates with a lower degree of confidence in the welfare state, greater interpersonal trust and greater belief in individuals and their capacity to secure their own well-being” (p. 511).

Likewise, there is a large difference in civic participation rates within Europe, where the majority of residents of the Nordic welfare system participate (67%), as opposed to those of the Mediterranean (27%) and Eastern (17%) systems. These low participation rates may be due to specific customs and values in these countries, such as the emphasis on strengthening family ties, characteristic of Southern and Eastern countries, which results in senior duties and family tasks (e.g. grandparents taking care of their grandchildren), thus limiting their scope for involvement in participatory activities. Moreover, low participation rates in Eastern countries could also be attributed to the forced “volunteering” in social, cultural and political causes of the socialist regime they experienced when they were younger, which could result in a low willingness on the part of older citizens to participate (Morawski et al., 2020).

On the other hand, the fact that countries with higher and lower levels of participation are the ones to show a stronger correlation between participation and well-being (and therefore, the ones that benefit the most from such participation) is contrary to what Morawski et al., (2020) have found about the inverted U-shape relationship between volunteering and quality of life. This may be due to their study including countries from various continents, while ours takes only European countries. In fact, our results are more similar to the study performed by Hansen et al., (2018), whose sample was also solely European. These authors assert that the association between volunteering and well-being is stronger in countries where volunteering is less prevalent and receives less institutional support.

Also, the fact that the correlation scores between civic participation and well-being are stronger in those welfare systems where well-being averages are lower, and vice versa, may be due to those who report low well-being being more susceptible to a larger increase through participation. Contrariwise, those who have already achieved a relatively high level of well-being do not report such large benefits from participating, similar to the famous “Easterlin Paradox” (Linxiang & Wei, 2020).

The results also show that macro variables at the national level have an important weight in determining the levels of health, happiness and satisfaction of older residents, as well as confirming the robustness of the effect that civic participation has on well-being. It would have been reasonable to expect the values of these indicators to increase as the Gross Domestic Product (GDP) grows, however, as the countries included in this paper are developed, the impact of an increase in GDP on well-being is practically null. This result echoes the paradox that, in many developed European countries, economic prosperity does not go hand in hand with the levels of happiness and satisfaction experienced (Huppert et al., 2005; Linxiang & Wei, 2020). This, in turn, highlights the importance of understanding which other factors contribute to improving subjective wellbeing.

In addition, wealth distribution plays an essential role. The results of the present study indicate towards a positive relationship between economic inequality and subjective health and life satisfaction. Much controversy and mixed results have been raised in the scientific literature regarding the relationship between income inequity and well-being (Yu & Wang, 2017). Authors Yu and Wang (2017) respond to this conflict by proposing that the relationship between inequality and happiness represents an inverted U-shaped curve, such that when disparity is relatively low, happiness levels rise because people see inequality as a sign of social mobility and expect upward progress for everyone. The authors also reported that, before the inflection point of the curve (as is the case for most European countries) where the level of income inequality is critical, a positive relationship between inequity and happiness is expected, probably because individuals compare themselves socially with their wealthier peers and consider that they too can achieve a higher financial status.

Furthermore, higher social expenditure also leads to lower levels of health, happiness and satisfaction, which could be due to the crowding-out effect (Bredtmann, 2016). This effect proposes that when a large proportion of essential services are covered by the state, citizens perceive that their participation is not indispensable, which may influence their subjective well-being by a perception that their contribution to public life is not of much value. Consequently, in the opposite case, in countries where public social expenditure does not cover social gaps, the feeling of need promotes citizens’ involvement, which enhances social capital as another form of welfare provision (Akaeda, 2021; Morawski et al., 2020).

McBride et al., (2006) affirm that “regardless of the form, there are consequences for individuals, communities, and representative democracy when citizens are not engaged” (p. 152) since this type of participation can be a way to improve the skills and capacities of individuals, but also of communities through increasing tolerance, representing interests that reinforce democratic governance and building community with shared support systems, objectives and actions.

Therefore, civic participation should be promoted through public and social policies. For such policies to be well targeted, and given that civic participation in turn is divided into various participatory activities, it is essential to determine which factors encourage engagement in such activities within the target society. For example, in the European context, Sánchez-García et al., (2022) recommend supporting senior volunteering through the provision of funds for social inclusion in solidarity networks, facilitating accessibility to the spaces where these activities take place, and improving support for the care of family members that often falls in the hands of the elderly. In the same way, these resources should be guaranteed so that seniors can engage in other participatory activities. Likewise, policies aimed at enabling diversity experiences are suggested, given that these are associated with greater civic attitudes, intentions and behaviors (Bowman, 2011). Furthermore, policies that promote the implementation of civic-oriented programs within the curricular and/or extracurricular activities of educational institutions (Bringle et al., 2011), so that their graduates develop life-long civic participation habits, are encouraged.

Finally, the hypothesis presented in this research would stand as follows:

**H1**: Rates of civic participation and well-being vary according to European welfare systems: Nordic countries are the most participatory and have the highest well-being levels, while Eastern Europeans are the least participatory and have the lowest well-being. **Mostly accepted: Nordic countries are the most participatory and have the highest levels of happiness and life satisfaction, but the Anglo-Saxon are the healthiest. Eastern European countries are indeed the least participatory and report the lowest well-being.**

**H2**: The effect of civic participation on well-being is stronger in countries with medium participation rates, and weaker in countries with high and low participation rates. **Rejected: results show that the relation between civic participation and well-being is stronger in countries with high and low participation rates, and weaker in countries with medium participation rates.**

## Limitations and Future Research

One of the limitations of this study is the cross-sectional nature of the data collected from the European Social Survey, such that when we refer to the relationship between civic participation and well-being, we do not claim causality. However, this shortcoming is mitigated by the fact that the present study is grounded upon earlier research, where the directionality of the relationships was analyzed by means of a pseudo-panel (Vega-Tinoco et al., 2021).

Another limitation corresponds to including only the 14 European countries that had data available for all waves (ESS 2002–2018) so that, when grouping them by welfare system, the Eastern and Mediterranean welfare systems comprise only 2 countries each, when in practice there are several other countries that fall into these categories and, if included, might modify the results.

Also, it was not possible to draw any conclusions about the results of the Eastern European regime in any of the multilevel regression models, since they were not significant. And, in addition, some of the means calculated in the descriptive statistics are smaller than the standard deviation, which indicates that the mean is not perfect for presentation and that the values within each welfare system vary greatly.

Given the significant differences in the rates of civic participation, future lines of research include an in-depth analysis of the determinants that motivate citizens to engage, or not, in this type of activities. Using a similar multilevel method, it may also be possible to study the factors that account for the differences in elderly civic participation among countries or welfare systems.

## Data Availability

all data have been drawn from the European Social Survey. The data are free of charge and publicly available on its official webpage.

## Footnotes

1 Within Model 3, interactions between civic participation and welfare systems were also conducted. However, only 3 out of 12 possible coefficients were statistically significant, thus being excluded from the model.
